# Supplementary material for: Identification of photoperiod-induced specific miRNAs in the adrenal glands of Sunite sheep (Ovis aries)
Source: Front Vet Sci. 2022 Jul 22;9:888207. doi: 10.3389/fvets.2022.888207 (PMC9354845; doi:10.3389/fvets.2022.888207)
Supplement: Supplementary file 1 [file Data_Sheet_1.ZIP › Supplementary file/miRDeep2 file.zip!/miRDeep2 file/Know_Structure/SPLP42A2/oar-mir-487a.pdf]

miRBase precursor : oar-mir-487a
Total read count : 127
oar-miR-487a-5p read count : 31
oar-miR-487a-3p read count : 94
remaining reads : 2

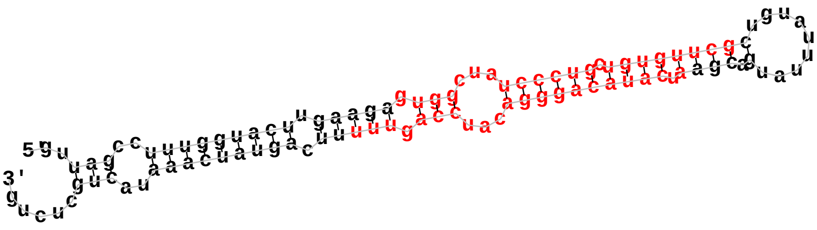

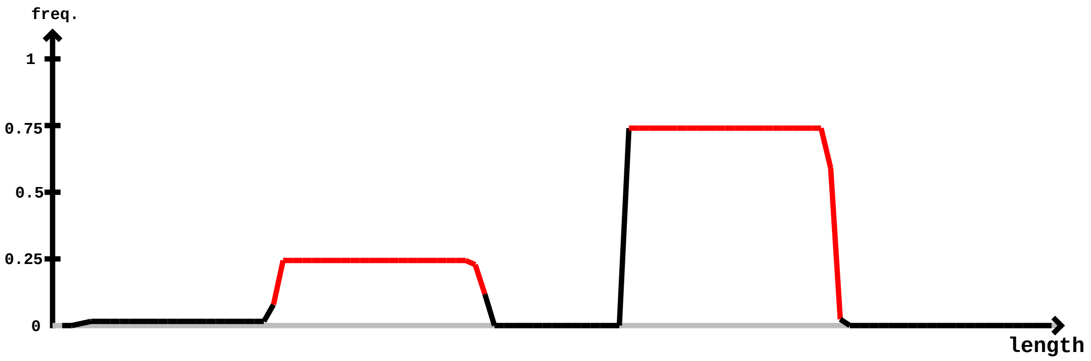


```
                                                             oar-miR-487a-3p
                          oar-miR-487a-5p
5'- guuagccuuugguacuugaagaguggcuaucccugcuguguucgcuguauuuaugacgaaucauacagggacauccaguuuuucaguaucaaauacugcucug   -3'    exp
    ..(((..(((((((((.(((((.(((...((((((.(((((((((.........).)))).))))))))))...))).))))).)))))))))..)))..... reads     mm        sample
    .uuagccuuugguacuugaag.................................................................................. 1         0         seq
    ..uagccuuugguacuugaaga................................................................................. 1         0         seq
    .....................aguggcuaucccugcuguguu............................................................. 2         0         seq
    .....................aguggcuaucccugcuguguuc............................................................ 6         0         seq
    .....................aguggcuaucccugcuguguucg........................................................... 1         0         seq
    ......................guggcuaucccugcuguguuc............................................................ 8         0         seq
    ......................guggcuaucccugcuguguucg........................................................... 14        0         seq
    ..........................................................aaucauacagggacauccagu........................ 19        0         seq
    ..........................................................aaucauacagggacauccaguu....................... 72        0         seq
    ..........................................................aaucauacagggacauccaguuu...................... 3         0         seq
```
